# Hyperbaric Oxygen Therapy: An Effective Auxiliary Treatment Method for Large Jaw Cysts

**DOI:** 10.7150/ijms.57360

**Published:** 2021-09-21

**Authors:** Dou Huang, Kaide Li, Xiaohui Zheng, Lei Liu

**Affiliations:** State Key Laboratory of Oral Diseases & National Clinical Research Center for Oral Diseases & Dept. of Oral and Maxillofacial Surgery, West China Hospital of Stomatology, Sichuan University, Chengdu, China.

**Keywords:** jaw cyst, hyperbaric oxygen therapy, bone substitute, infection, bone repair

## Abstract

**Background:** To evaluate hyperbaric oxygen therapy (HBOT) on infection rates and repair rates during the treatment of large jaw cysts.

**Methods:** A prospective randomized, non-blinded, controlled clinical trial included 90 patients with jaw cysts, randomly divided into three groups. Patients were treated with enucleations and bone substitute was used in the experimental and control groups. The experimental group received HBOT. The primary predictor variable was HBOT. The infection rate, repair rate, preoperative volume of the jaw cysts, age, and sex were statistically analyzed. The Fisher exact test was used to compare the infection rate and postoperative complications. The repair rate of the bone defects was analyzed using the repeated-measures analysis of variance and the least significant difference tests. The Kendall's coefficient of concordance and Kappa statistics were calculated to evaluate the consistency between the two investigators.

**Results:** The infection rate was 3.4% in the experimental group, 14.3% in the blank group, and 32.1% in the control group (P<0.05). The repair rate in the experimental group was significantly higher than in the control and blank groups at 1, 3 and 6 months after surgery (P<0.05).

**Conclusion:** The results showed that HBOT reduced the postoperative infection rate following the enucleation of large jaw cysts with bone substitute filling, and it also improved the bone repair rate.

## Introduction

Jaw cyst is a common disease that can cause expansion, destruction and infection of maxillofacial bones, significantly impacting patients' quality of life. Studies have reported that in oral and maxillofacial pathology practices, jaw cysts are present in 14%-17.5% of specimens 1-2. The treatment of jaw cysts includes enucleation, marsupialization and bone resection 3. Enucleation is the most common procedure as the cysts are removed whole, with less discomfort and inconvenience for the patients 4, 5. Occasionally, in large jaw cysts, marsupialization and bone resection are the chosen treatment modality 6, 7. Marsupialization maximizes the preservation of the tissue; however, it requires high patient compliance owing to the long treatment duration and a second enucleation in most cases 8. Furthermore, bone resection is a radical operation as it can destroy the bone contour, influence the physiological functions of the jaw, and decrease the patients' quality of life 9. Therefore, enucleation is still the most widely applied surgical treatment for jaw cysts.

Unfortunately, clinical practice has shown that bone defects left following enucleation, especially with large cysts, are hard to heal 10. The bone defects are likely to form a dead cavity that carries risks, including infection, soft tissue depression and even pathological fracture 5. Many reports have shown that bone substitutes are a promising solution by promoting ossification and preventing tissue depression 11, 12. However, further studies and clinical trials have shown that there is an increased risk of infection for the patients treated with bone substitute 13, 14. It is possible that the bone substitute provides a site for bacteria to evade the host immune surveillance especially in the central area of the defect 15. Once infection occurs, a second surgery is often needed to remove the bone substitute, prolonging the therapy period and with great discomfort for the patients. Some studies have been suggested that bone substitutes should not be used with large jaw cysts 5,16. Therefore, the large bone defects occurring after enucleation are still a huge challenge for surgeons.

Hyperbaric oxygen therapy (HBOT) is a kind of treatment whereby patients inhale 100% oxygen at super-atmospheric pressure and has been widely used in refractory diseases, osteomyelitis, compromised skin graft and flap, anaerobic and so on 17. There are many studies that showed that HBOT could inhibit the growth of anaerobic bacteria, ease inflammation, and promote ossification 18, 19. Therefore, we propose that HBOT could help to reduce the risk of postoperative complications, as well as promoting ossification after enucleation. To date, there is no report on the application of HBOT combined with bone substitute filling cystic cavities. Therefore, we explored the use of HBOT for the treatment of large jaw cysts.

The purpose of this study was to evaluate whether HBOT can effectively decrease the risk of postoperative complications and accelerate the ossification in patients with large jaw cysts. We hypothesized that HBOT could reduce the risk of postoperative infection and promote ossification. The specific aim of the study was to compare the infection rate and repair rate of the bone defect in three groups.

## Materials and methods

### Study design and patient samples

The authors designed and conducted a prospective, randomized, non-blinded, controlled clinical trial that was approved by the institutional research board of our institute (review document: WCHSIRB-CT-2020-229). The authors read the Declaration of Helsinki and followed its guidelines in this study.

Jaw cysts are cystic lesions occurring in the upper and lower jaws, which can be classified as inflammatory or developmental depending on the pathogenesis; as true cysts or pseudocysts, depending on whether there are lining epithelial and odontogenic or non- odontogenic depending on the tissue of origin 20. The patients included in this study were diagnosed with a single jaw cyst by two clinically experienced doctors and gave consent for the surgical treatment. The patients whose longest cystic diameter was greater than 2 cm on computerized tomography (CT) images were enrolled in the study 5,20. The patients were excluded from this study if they refused surgical treatment; they could not complete at least 6-month follow-up; they could not receive HBOT for 30 days; they were minors, pregnant women or suffered mental disorders.

The values of α = 0.05, power (1 - β) = 90%, the infection rate was 40% in the control group, and was 5% in the experimental group based on the references and suggestions of clinical specialists. Then, the number of patients in each group was calculated using NCSS-PASS 15 software. We found that 25 patients in each group were necessary for this study. Considering potential dropouts, we expanded this number by 20% and the final sample size was 90 patients.

This study included 90 patients with maxillofacial bone cysts undergoing enucleations between September 2016 and June 2019 in our institution. The patients were randomly divided into three groups according to a random numbers table, including the experimental group, control group and blank group, with 30 in each group. All patients were treated with enucleations. The bone substitute was used to fill in both experimental and control group patients, while the 30-day HBOT was applied only in the experimental group. Neither bone substitute nor HBOT was applied in the blank group.

### Material

The HBOT was performed in a hyperbaric chamber (HAUX, Karlsbad-Ittersbach, Germany). The bone substitutes were β-tricalcium phosphate (DePuy Synthes, Oberdorf, Switzerland). The guided tissue regeneration (GTR) membranes were bovine acellular dermal matrix (Zhenghai, Qingdao, China). All preoperative and postoperative imaging data were obtained using a Brilliance CT apparatus (Philips, Best, the Netherlands), and the data were imported into Mimics 16.0 (Materialise, Leuven, Belgium). SPSS 23.0 (IBM Corp, New York, USA) and NCSS-PASS 15 software (NCSS, Utah, USA) were used for statistical analyses.

### Study variables

The primary predictor variable was HBOT. The primary outcome variables were the infection rate and the repair rate of the bone lesion between preoperative and postoperative CT measurements after 1, 3 and 6 months. Other variables related to the study were preoperative volume of the jaw cysts, the amount of artificial bone, age, sex, and other postoperative complications.

### Surgical methods

After administering general anesthesia with nasotracheal intubation, all patients were operated on by one experienced surgeon. Different incisions were chosen according to the location of the lesion, and intraoral incision was the most common approach. After the periosteum was stripped from the bone surface, part of the lateral bone wall was removed to expose the cystic tissue. Subsequently, the cysts were removed whole, and the defect was rinsed with normal saline solution (0.9% sodium chloride). The β-tricalcium phosphate was implanted into the bone defects, and the GTR membrane was placed and fixed to cover the lateral wall of the bone defect in the experimental group and the control group but not in the blank group. Finally, the wound was sutured.

Following the surgery, the patients in the experimental group were required to receive HBOT for 30 days, while the patients in the control group and blank group did not receive HBOT. The HBOT therapeutic regimen was a half past one hour treatment of breathing 100% oxygen once a day for 30 days in a hyperbaric chamber pressurized to 2.0 atmosphere absolute.

The wounds were examined daily for 7 days before the sutures were removed. When the infection occurred, incision, drainage and antibiotic therapy were performed, and the surgical debridement was performed if necessary.

### Data collection and measurement

Postoperative clinical and laboratory characteristics were observed, graded and recorded from day 1 to day 7, including swelling, skin color and temperature, pain, wound secretions, fever etc. The two surgeons determined whether there was the infection. Postoperative follow-up visits were set at 1, 3, 6 and 12 months and CT images, the complications were observed and recorded. Clinical follow-up and evaluations were performed by two surgeons who were on the surgical team. If there was a depression of more than 3 mm in the operative area, the soft tissue depression was considered to occur. Radiologic analyses were performed by two doctors who were not on the surgical team. The preoperative volume of the cyst and the residual low-density areas in the bone defect at 1, 3, 6 months after the operation were calculated with Mimics 16.0 software. The differences between the residual low density areas at 1, 3, 6 months and the preoperative volume of the cyst were calculated respectively. The ratio of the difference to the preoperative volume of the cyst was the bone repair rate.

### Statistical analysis

Two doctors who were not on the surgical team undertook the statistical analysis. The Fisher exact test was used to compare the infection rate and postoperative complications. The repair rate of the bone defects was analyzed using the repeated-measures analysis of variance and the least significant difference tests. The Kendall's coefficient of concordance (W) and Kappa statistics were calculated to evaluate the consistency between the two investigators. All statistical analyses were calculated using SPSS 23.0 for Windows. A P value less than 0.05 was considered statistically different.

## Results

Overall, 90 patients were enrolled in the study, of which 1 patient did not complete the 30-day HBOT and 4 patients did not complete the 6-month follow up. Therefore, 85 patients completed the study with 29 patients in the experimental group, 28 patients in the control group and 28 patients in the blank group. The total number of patients included 49 men and 36 women, with a mean age of 40.15 years (range 18-69 years). There were 21 cysts in the left maxilla, 20 in the right maxilla, 21 in the left mandible and 23 in the right mandible. The operations and postoperative care were well performed in all patients. Histological examinations after the operations reported that there were 42 dentigerous cysts, 20 odontogenic keratocysts, 21 radicular cysts and 2 nasopalatine duct cysts. HBOT was conducted successfully in the experimental group. The follow-up periods varied from 6 months to 12 months, and the mean period was 9.4 months.

A comparison of infection rate among the 3 groups is presented in Table 1. The infection rate was 3.4% in the experimental group, 14.3% in the blank group, and 32.1% in the control group (P<0.05). These results indicated that the infection rate in the experimental group was lower than that in the control and blank groups (P<0.05). Of all the infected patients, 1 patient in the experimental group, 8 patients in the control group and 1 patient in the blank group were performed surgical debridement. The infections of 1 patient in the control group and 3 patients in the blank group were controlled through incision and drainage.

A comparison in the repair rate of the bone defect at each time point is presented in Table 2. There were the 28 patients without infection in the experimental group, 19 in the control group and 24 in the blank group. Of these patients, the repair rate in the experimental group was significantly higher than in the control group at 1, 3 and 6 months after the surgery (P<0.05). In the intra group comparison, the bone repair rate of each group was the lowest in 1 month after the surgery and the highest in 6 months after the surgery (P<0.05, W=0.712-0.868). These results indicated that the repair rate in the experimental group was the highest compared with the control group and blank group.

A comparison of other variables was showed in the Table 3. The rate of soft tissue depression was 35.71% in the blank group, 10.7% in the control group and 0% in the experimental group (P<0.05, κ =0.849). However, there was no statistical difference in the three groups regarding the preoperative volume of the jaw cysts, age, sex, and other postoperative complications.

## Discussion

The infection of large bone defects filled with bone substitute after enucleation is still a serious issue in the treatment of large cysts 13. Therefore, a new and effective treatment method is much needed, and HBOT has been suggested as a promising solution 17-19. Therefore, we conducted a randomized controlled clinical trial to assess the effects of HBOT during the treatment of patients whose bone defects were filled with bone substitute after enucleation.

In the present study, the results showed that the postoperative infection rate was lower in the experimental group than that in the control and blank groups. The infection rate was 3.4% in the experimental group, 14.3% in the blank group, and 32.1% in the control group. These results indicate that the bone substitute is a risk factor of infection and HBOT plays a role to reduce the postoperative infection rate. Mechanisms of action might be that HBOT could increase the local oxygen partial pressure, directly killing or inhibiting bacteria, especially anaerobic bacteria, which are the most common pathogenic bacteria in oral and maxillofacial infections. HBOT may also boost the function of the immune system, particularly the bactericidal effect of leukocytes, or it may enhance the function of antibacterial drugs 21,22.

The repair of bone defects left after enucleation is problematic in clinical practices. In this study, the CT data showed that the repair rate in the experimental group was the highest among the three groups at the 1, 3 and 6 months after the operation, and that in the experimental and control group was much higher than in the blank group. These results indicate that the bone substitute can greatly give assistance to bone repair and HBOT can accelerate osteogenesis. Possible mechanisms for promoting ossification by HBOT include enhancing the osteogenic activity on human osteoblasts and bone marrow mesenchymal stem cells; stimulating neovascularization; and increasing the accumulation of calcium, magnesium, phosphorus and other minerals needed for osteogenesis 23-24.

There were several limitations to this study, such as the small samples and results from only a single center. As a result, selection bias and confounding bias could exist. This study suggested that HBOT could reduce the infection rate, as well as promote bone healing after enucleation; however, a larger scale, multicenter clinical study is needed to confirm the practical value of HBOT in the treatment of jaw cyst.

In conclusion, the results showed that the use of HBOT was helpful to reduce the postoperative infection rate after the enucleation of large jaw cysts with bone substitute filling, and it also improved the bone repair rate. Therefore, HBOT should be considered for application in clinical work.

## Figures and Tables

**Table 1:** The infection rate of the three groups

	Experimental Group (29)	Control Group (28)	Blank Group (28)	P value
Infection (n, %)	1 (3.4%)*	9 (32.1%)	4 (14.3%)	0.012
No infection (n, %)	28 (96.6%)*	19 (67.9%)	24 (85.7%)	

Note: Chi squared test (Fisher exact probability method) was used; *: Experimental group and control group differed significantly at the P < 0.05/3 = 0.0167; (Bonferroni adjustment) level.

**Table 2:** The repair rate at 1, 3 and 6 months in the three groups

Repair rate at	Experimental Group (N=28) (Mean±SD%)	Control Group (N=19) (Mean±SD%)	Blank Group (N=24) (Mean±SD%)	*P Value
1^st^ month	86.39±2.02	84.26±1.95	8.04±2.05	.001
3^rd^ month	90.18±3.21	86.02±1.82	14.09±2.63	.000
6^th^ month	96.32±2.50	90.38±1.51	21.73±4.03	.000
**P Value	.000	.000	.000	

Note: The repeated-measures analysis of variance and least significant difference tests for pairwise comparisons were used for analysis. *: Group effect P value (1^st^ vs. 3^rd^ vs. 6^th^ month). **: Time effect P value (Experimental vs. Control vs. Blank group).

**Table 3:** The comparison of patient characteristics and postoperative complications

Study Variables	Experimental Group	Control Group	Blank Group	P Value
Sample size, n	29	28	28	A
Age years (mean±SD)	45.2±26.4	41.6±20.8	43.9±24.3	0.64
**Gender, n**				0.09
Men	15	18	16	
Women	14	10	12	
Mean volume of cysts preoperatively (mm³)	5230.3±2025.6	4831.9±1789.5	4761.3±1864.2	0.17
Relevant nerve injury, n (%)	0 (0)	0 (0)	0 (0)	1
Soft tissue depression, n (%)	0 (0)	3 (10.7)	10 (35.7)	.000
Pathological fracture, n (%)	0 (0)	0 (0)	0 (0)	1
**Histology, n (%)**				0.891
Dentigerous cyst	12 (41.4)	14 (50)	16 (57.1)	
Odontogenic keratocyst	8 (27.6)	7 (25)	5 (17.9)	
Radicular cyst	8 (27.6)	6 (21.5)	0 (0)	
Nasopalatine duct cyst	1 (3.4)	1 (3.5)	0 (0)	
**Sites, n (%)**				0.666
Left maxilla	9 (31.1)	6 (21.4)	6 (21.4)	
Right maxilla	7 (24.1)	4 (14.3)	9 (32.2)	
Left mandible	6 (20.7)	8 (28.6)	7 (25.0)	
Right mandible	7 (24.1)	10 (35.7)	6 (21.4)	

Note: Chi-squared test (Fisher exact probability method) and analysis of variance were used for analysis.
